# Pd-Ag Electrical Resistivity in Hydrogen and Deuterium: Temperature Effect

**DOI:** 10.3390/ma12213551

**Published:** 2019-10-29

**Authors:** Alfonso Pozio, Silvano Tosti

**Affiliations:** 1 Department of Energy Technologies, ENEA C.R. Casaccia, Via Anguillarese 301, S. Maria di Galeria, 00123 Rome, Italy; 2 Department of Fusion and Technology for Nuclear Safety and Security, ENEA, C.R. Frascati, Via E. Fermi 45, 00044 Frascati, Italy; silvano.tosti@enea.it

**Keywords:** Pd-Ag alloy, hydrogen, deuterium electrical resistivity

## Abstract

The electrical resistivity of Pd-Ag (silver 21 wt.%) in hydrogen and deuterium atmosphere at 100 kPa has been investigated via electrochemical impedance spectroscopy. The electrical resistivity of Pd-Ag vs. the temperature presents the characteristic S-shaped curve with a minimum and a maximum of the resistivity in different positions for the tests in hydrogen and deuterium. The results have been related to: (1) the different isotope ratios, H/M and D/M, and (2) their position in the Pd-Ag lattice. The behavior of the electrical resistivity is discussed in details by considering the hydrogen and deuterium uploading into the alloy, its effect on the conduction electrons, and the scattering of the isotopes atoms into the metal lattice. Measurements carried out in hydrogen with slow temperature ramping between 25–250 °C evidenced a hysteresis effect that can be explained by the different energy levels of isotopes in O-sites and T-sites

## 1. Introduction

Palladium-based membranes are studied for technological processes such as the separation and purification of hydrogen [1,2]. Potential applications concern the fusion fuel cycle where hydrogen isotopes have to be separated from plasma exhausts, from breeding blankets, and from coolants (water, helium) [3]. Several methods are proposed for recovering tritium from water [4,5,6,7,8,9] and, in particular, the use of membrane processes introduces the advantages of high reliability and continuous operations, ensuring the more efficient and safe management of a fusion power plant [10].

The uploading of hydrogen into pure Pd can involve metal embrittlement. In fact, at atmospheric pressure and below 300 °C, the hydrogen is absorbed into Pd in two hydride phases (α and β) characterized by different values of the lattice parameters (0.3894 and 0.4025 nm, respectively): the transition between these two hydride phases involves cyclic strains of the lattice that are responsible for the embrittlement of hydrogenated Pd. The alloying of Pd with Ag [11,12] reduces the coexistence of the hydride phases α and β, thus reducing the hydrogen embrittlement. Ag alloying also improves the mechanical properties and hydrogen permeability [13]; both the tensile strength and the permeability show a maximum for silver content in the range 20%–40% of weight that is the Ag content of commercial Pd-alloys used for membrane applications. Also of note, the electrical resistivity of non-hydrogenated Pd-Ag alloys presents a maximum in the range 20–40 wt.% of Ag loading [14]. Such a feature is of interest when the direct ohmic heating of Pd-permeators is deemed necessary. In this case, the temperature is controlled by powering the ends of a thin-walled Pd-Ag tube with electrical current [15]: the higher the electrical resistivity of the Pd-alloy, the lower the electrical current to be fed through these permeator tubes will be. The membrane units realized in this way make it possible to reduce the energy consumption and to speed up the heating, compared to conventional membrane modules [15]. Several literature works concern the effect of hydrogen isotopes on the properties of different Pd-alloys. These data are addressed especially on the analysis of mass transport parameters (diffusion, solubility, and permeability) in order to design process units for separation of hydrogen isotopes.

Kimura et al. [16] investigated the permeation of H_2_ and D_2_ through a Pd-Ag alloy in a low concentration range where the formation of HD was negligible. Mitsuishi et al. [17] studied the permeation of H_2_, HD, and D_2_ for H_2_-Ar, D_2_-Ar, and H_2_-D_2_-Ar systems through a Pd-Ag-Au alloy tube at 400 °C. Luo et al. [18] measured isotherms for H_2_/D_2_-Pd_0.8_Ag_0.2_ over a low temperature range, −75 to 50 °C. The H/D isotopic effect and the separation factors in the plateau range have been calculated and discussed along various isotherms. 

Nishikawa et al. [19] performed permeation of hydrogen isotopes through a Pd_0.75_-Ag_0.25_ membrane in the temperature range 80 to 400 °C using the co-current double tube method by assessing the isotopic effect in permeation. The results showed that permeation is limited by diffusion of hydrogen atoms in the bulk of the membrane. Ackerman et al. [20] evidenced deuterium permeation lower than the hydrogen one through Pd_0.75_-Ag_0.25_ silver alloy tubes at driving pressures up to 100 kPa and at temperatures of 300, 400, and 500 °C. 

Lasser et al. [21] evaluated the solubility of protium, deuterium, and tritium in palladium-silver alloys at low hydrogen concentrations. Furthermore, the ground state energies of hydrogen isotopes dissolved in these alloys have been determined. Paolone et al. [22] measured the hydrogen and deuterium solubility up to 7 bar for Pd_0.77_Ag_0.23_ in the temperature range between 25 °C and 400 °C. In this work, the occurrence of solid solutions or hydride phases has been discussed and the hydrogenation enthalpy has been calculated. Anand et al. [23] measured equilibrium pressure-composition-temperature (PCT) relationships for Pd_0.77_-Ag_0.23_ over the temperature range of 101–146 °C, evaluating thermodynamic parameters like enthalpy and entropy of hydrogen/deuterium desorption reaction. Hydrogen/deuterium absorption for this alloy was investigated in the temperature range of 62–121 °C, and activation energies of hydrogen/deuterium absorption reactions were evaluated. Both thermodynamic and kinetic data showed pronounced normal isotopic effect in the temperature and pressure ranges investigated.

Hickman [24] measured solubility and permeability coefficients of deuterium in Pd_0.75_-Ag_0.25_ between 0 and 6900 kPa at 300, 400, and 500 °C. Deviations from ideal behavior have been observed, particularly for diffusion coefficients, so that permeability cannot be represented as a simple function of pressure. The non-ideality was attributed to the lattice expansion at high D/M ratios. A strain-induced shift in solubility was also observed. Despite the large amount of works on this alloy, no data concerning the isotopic effect on electrical resistivity has been found in the literature. Few works exist on the effects of the Pd/hydrogen isotope interactions on electrical resistivity [25,26].

The resistivity of a Pd_1−x_-Ag_x_ alloy in hydrogen has been studied in the past, showing a typical S-shaped curve as a function of x (wt.%), appearing for x = 10 and disappearing completely at x = 50 [27,28,29].

In a previous work [30], the electrical resistivity of hydrogenated Pd-Ag (silver 21 wt.%) has been measured in the temperature range 25–350 °C via electrochemical impedance spectroscopy. At a given hydrogen pressure, the electrical resistivity vs. the temperature exhibited the characteristic S-shaped curve with a minimum and a maximum of the resistivity. Such a behavior has been discussed, evaluating both the effect on the conduction electrons and the scattering of the H atoms in the metal lattice. Now, in the present study, we have extended electrochemical impedance spectroscopy to deuterium. The electrical resistivity vs. the temperature in deuterium presents similar S-shaped curves with the minimum and maximum of the resistivity in different positions. The behavior of the electrical resistivity has been discussed in details by considering the hydrogen and deuterium uploading, its effect on the conduction electrons, and the scattering of the isotope atoms into the metal lattice. Particularly, hydrogen occupancy of octahedral and tetrahedral sites (responsible for inverse and normal isotopic effects, respectively) is characterized by different energy levels that could explain the hysteresis of the resistivity curve observed at slow temperature ramping between 25–250 °C.

## 2. The Isotopic Effect on Pd-Ag Resistivity and Solubility

To discuss experimental results, we must consider the isotopic effect on solubility and resistivity of hydrogenated Pd-Ag. In the first approximation, the H and D atoms in a metal lattice behave like independent simple harmonic or “Einstein” oscillators. Hydrogen and deuterium substitution can affect the vibrational frequency of the chemical bond that they form with the lattice, even if the potential energy surface for the reaction is nearly identical. Heavier isotopes (classically) lead to lower vibration frequencies or, viewed quantum mechanically, have lower zero-point energy (ZPV) [31]. The difference in zero-point vibration energies, E_vib_, between H and D is most likely to be the cause of isotopic effects. In the quantum harmonic oscillator approximation, vibrational energy is described by the Equation [32]:

(1) $$E_{\mathrm{vib}}=\left(n+\frac{1}{2}\right)h\nu_{0}=\left(n+\frac{1}{2}\right)\frac{h}{2\pi }\sqrt{\frac{K_{F}}{\mu }}$$

where *n* represents the energy level, *h* is the Planck’s constant, *υ*_0_ is the normal frequency of vibration, *K_F_* is the force constant, and *μ* is the reduced mass.

**Figure 1:**
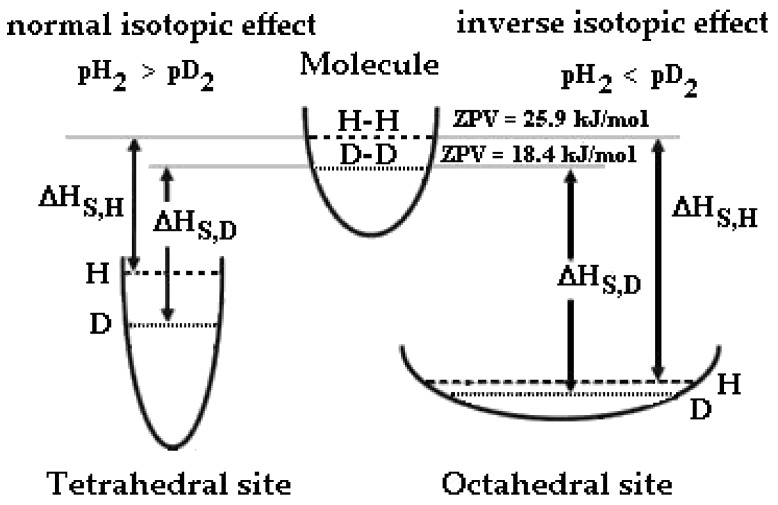
Schematic description of potential wells and zero-point vibration energies of H and D in a H_2_ and D_2_ molecule, octahedral and tetrahedral sites of Pd-Ag and isotopic effect on the enthalpy of solution, ΔH_S_.

The frequency of the oscillations of the atoms can also be expressed using the Einstein temperature [33]:

(2) $$\theta_{E}=\frac{hv_{0}}{k_{B}}$$

where *k_B_* is the Boltzmann constant. Therefore, the lighter hydrogen isotope has the higher vibrational energy, E_vib_(H) > E_vib_(D), or the higher *θ*_E_. With a lower zero-point energy (ZPV, E_vib_ for *n* = 0), more energy must be supplied to break the bond, resulting in a higher activation energy for bond cleavage (Figure 1). As an example, to dissociate H_2_, it needs an energy of 431.7 kJ/mol instead of 439.2 kJ/mol for D_2_. When the only contribution was the loss of the zero point energy difference (i.e., complete failure of the bond), the expected isotopic effect should be about 1.41, having a frequency of vibration, *υ*_0_, of 4370 cm^−1^ for H_2_ and 3091 cm^−1^ for D_2_.

We should consider the energy levels of deuterium and hydrogen inside the Pd-Ag lattice. Generally, in metals with a face-centered cubic structure (FCC) such as Pd-Ag hydrogen, isotopes occupy mainly octahedral sites (O-sites), as the volume space of O-sites is larger than that of T-sites [34,35].

Since the *K_F_* of hydrogen isotopes in O-sites is smaller than that in T-sites, the shape of the potential wall in the O-sites is smooth (Figure 1). The different potential energy profiles give rise to a very different ZPV of hydrogen in T-sites (~150 meV) with respect to octahedral site (~60 meV) [36,37,38].

Now, we should consider that the absolute level energy for the enthalpy follows the trend |ΔH_S,H_^O^| > |ΔH_S,D_^O^| > |ΔH_S,D_^T^| > |ΔH_S,H_^T^|, for this reason, different equilibria can occur when we upload hydrogen isotopes into a metal at different temperatures. It is noteworthy that the absorption enthalpy is negative, so for the hydrogenated Pd-Ag the lowest enthalpy is that of H in O-sites. In order to highlight the different isotopic behavior into a Pd-Ag lattice, we can establish that: (1) the absolute enthalpy solution of both isotopes in the O-sites is lower than in T-sites, and (2) in O-sites, the absolute enthalpy solution of hydrogen is lower than the deuterium one (and vice versa for T-sites). 

At room temperature and high pressure (namely, high hydrogen uploading levels), all sites T and O of the Pd-Ag lattice are occupied so that no significant differences of chemical-physical properties can be observed between the hydrogenated and deuterated metal. By increasing the temperature (i.e., by providing thermal energy to the system), first, hydrogen leaves the T-site, having the lowest enthalpy, followed by deuterium from the T-site, then deuterium from the O-site and finally hydrogen from the O-site.

According to the definition given in the literature [22,36], when tetrahedral sites are occupied (low temperature), the Pd-Ag-H system exhibits a normal isotopic effect (i.e., the equilibrium pressure of hydrogen isotopes in gas and solid phases is pH_2_ > pD_2_), while at high temperature (octahedral occupancy), an inverse isotopic effect occurs (pD_2_ > pH_2_). In general, Pd-Ag is expected to exhibit an inverse isotopic effect due to the occupancy of the O-sites; however, at high uploading (low temperature and high pressure), the T-sites are also occupied and the Pd-alloys shows a normal isotopic effect. In practice, the Pd-Ag alloy exhibits a completely different behavior when hydrogenated at low or high H/M (D/M) ratios: similar behavior (occupancy of O- and T-sites with inversion of the isotopic effect) has been observed for V-H and Ti-H systems [36].

Vacuum experiments dedicated to measuring the hydrogen absorption temperature [39] confirmed that, by increasing the temperature, the equilibrium tetrahedral ↔ octahedral shifts toward the right. We can presume that the same equilibrium exists for deuterium:

(3) $$H_{T}↔H_{O}$$

(4) $$D_{T}↔D_{O}$$

where subscripts indicates O e T-sites.

In particular, we can observe that the difference, |ΔH_S,H_^O^| − |ΔH_S,H_^T^|, is higher than |ΔH_S,D_^O^| − |ΔH_S,D_^T^| and, therefore, the presence of the hysteresis phenomena (H or D occupancy, resistivity, etc.) should be observed through a slow adsorption/desorption (i.e., under quasi-equilibrium conditions) of hydrogen and deuterium. Indeed, heating and cooling hydrogenated Pd-Ag means a shift in equilibrium (3) and (4), but being that thermal energy for hydrogen adsorption/desorptionm is higher than that of deuterium, any hysteresis should be higher for hydrogen. This phenomenon will be presented in the last part of the Results section.

In order to discuss the isotopic effect on H and D solubility, it is opportune to define some parameters. The atoms of hydrogen isotopes per atom of metal, H/M (D/M) ratio, can be calculated from the solubility *s* (mol m^−3^) with the relationship [29]:

(5) H(D)M=2 ×s×PMPd−Agd×0.79

where *PM_Pd-Ag_* is the molecular weight of the alloy, *d* is the Pd-Ag 21 wt.% density (11,600 kg m^−3^), and 0.79 is the atomic percent of Pd into the Pd-Ag alloy.

Under equilibrium conditions, the hydrogen (or deuterium) solubilized in the metal, *s*, and its partial pressure, *p* (Pa), in the gaseous phase is expressed through Sieverts’ law by the equation [19,40,41]:

(6) $$s(H_{2})=K_{H}p^{0.5}$$

(7) $$s(D_{2})=K_{D}p^{0.5}$$

where *K_H_* and *K_D_* (mol m^−3^ Pa^0.5^) are the solubility constants for the two isotopes. They are a function of temperature according to an Arrhenius-type equation:

(8) $$K_{H}=A\times e^{B/RT}=0.182\exp(\frac{19598}{RT})$$

(9) $$K_{D}=A\prime \times e^{B'/RT}=0.184\exp(\frac{18531}{RT})$$

where *A*, *A*’, *B*, *B*’ are empirical constants obtained by gas permeation experiments [19,40,41], *R* = 8.314 J K^−1^ mol^−1^, and *T* is the temperature (*K*). From the above equations, at constant pressure we can write the ratio β(H/D) of solubilities s(H_2_)/s(D_2_) of hydrogen isotopes through Pd-Ag membranes as [19,40,41]:

(10) $$\beta (H/D)=0.989\exp(\frac{1067}{RT})$$

This expression calculates the D/M ratio from the corresponding H/M when Sieverts’ law is valid (i.e., pressure lower than 100 kPa and temperature higher than 200 °C). In contrast, at 100 kPa and a temperature lower than 200 °C, Sieverts’ law cannot be applied and Equations (6) and (7) assess solubility values lower than those experimentally measured. In the literature, the analysis of pressure composition isotherms [18,21,22,23] showed a sloping-plateau region distinct for H_2_ and D_2_. It is also evident that deuterium equilibrium pressure is higher than that of hydrogen at all experimental temperatures because of an inverse isotopic effect (pD_2_ > pH_2_). At the same time, all measurements showed that at high pressure, ≥ 100 kPa, as the temperature is reduced towards room temperature the difference of H/M and D/M reduces also.

In this study, both H/M [22,30,40,42,43,44] and D/M [18,21,22,23,31,45] were extrapolated by literature data on the same kind of alloy in order to compare solubility with resistivity data. The literature evidenced that at 100 kPa for low temperatures (25–70 °C) the D/M approaches the H/M value, while increasing the temperature (75–200 °C) causes the D/M to decreases faster, and only at high temperature (>300 °C) do both curves (H and D) tend to converge to very low values (close to zero).

As introduced above, the isotopic mass affects not only the concentration of hydrogen/deuterium into the metal lattice at a given temperature and pressure but also the composition-temperature boundaries between solid phases. The physical reasons for the effect of isotopic mass on phase boundaries are related to differences in the zero-point energies of the dissolved isotopes, the differences in the lattice expansions produced by the isotopes, and the differences produced in the phonon spectrum [32,46].

Electrical resistivity is also influenced by these effects because it depends on phonon scattering that is strictly related to the vibrational energy of isotopes in T- and O-sites [33,47]. Solids as Pd-Ag can exhibit two types of phonon scattering: acoustic phonons and optical phonons. Acoustic phonons are coherent movements of atoms in the lattice out of their equilibrium positions. Optical phonons are out-of-phase movements of the atoms in the lattice (i.e., one atom moving to the left and its neighbor to the right). Electron scattering centers are produced in metal hydrides when interstitial vacancies are occupied by H or D, producing a new optical phonon band. Smith et al. [26] observed that, for similar concentration of isotopes, Pd-H shows lower resistivity than Pd-D. They justified this behavior by claiming that a major change to the phonon spectrum with the addition of deuterium is the introduction of a band of optic modes. Tsuchiya et al. [48] and Bickel et al. [49] also observed lower electrical resistivities of hydrogenated ε-Zr at 25 and 325 °C: such a behavior was considered to be related to the electron-phonon scattering, where the optical mode plays an important role. 

## 3. Experimental Methods

The resistivity measurements were performed in four-electrode AC impedance mode using a Solartron 1260 frequency response analyzer by Schlumberger. The spectra were recorded between 1 Hz and 150 kHz with 10 points per decade and a maximum perturbation amplitude of 0.5 mA.

The metal sample consisted of a 21% by weight Pd-Ag thin strip of 141.0 mm length, 4.4 mm width, and 51 µm of thickness: it was obtained by the cold rolling of a commercial sample (Good Fellows). This sample had been located in a gas-tight stainless steel vessel while four parallel platinum electrical contacts were welded to the ends of the metal strip. In particular, the length (l) of the Pd-Ag strip between the voltage measurement points was 137.5 mm. The impedance in the tangential direction (in the plane) of the lamina was measured, and its resistivity was obtained using the equation:

(11) $$\rho =\frac{R_{\Omega }\times A}{l}$$

where *ρ*, *l*, *R*_Ω_, and A denote the resistivity (Ω m), the distance between the probe (m), the measured resistance (Ω), and the cross-sectional area (m^2^) of the sample Pd-Ag, respectively. The area, *A*, was calculated from the product of the thickness of the Pd-Ag strip (*r*) by its width (*h*).

**Figure 2:**
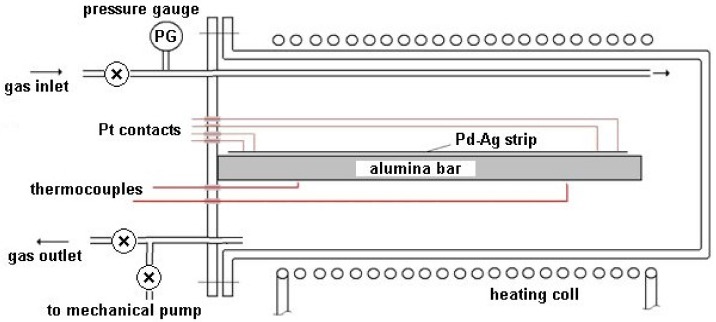
Experimental apparatus.

The sealed stainless steel module, provided with a gas inlet and outlet, was equipped with an electric heating apparatus based on temperature measurements carried out by two thermocouples (Figure 2). The module was thermally insulated and reduced in size in order to guarantee a homogeneous temperature along the entire volume.

Values of temperature and resistivity were recorded continuously during the tests. The experiments focused on the study of the electrical resistivity behavior of hydrogenated and deuterated Pd-Ag. The experimental campaign has been carried out in the range 25–450 °C under Ar, H_2_, and D_2_ atmospheres at 100 kPa. The first experiments were performed with a heating ramp of 5 °C/min, while the following tests were at 1 °C/min.

## 4. Results and Discussion 

### 4.1. Electrical Resistivity vs. Temperature

The dependence of the electrical resistance of metals on temperature, *ρ_T_*, is generally described by the linear equation [50]:

(12) $$\rho_{T}=\rho {}^{\circ}\;[1+\alpha \;(T-T{}^{\circ})]$$

where *T* and *T*° are the temperature and the reference temperature (usually room), *ρ°* is the resistivity at reference temperature *T*°, and α is the so called thermal coefficient (the variation of resistivity per unit of temperature).

For a limited temperature range, the value of α can be assumed in the first approximation to be constant and dependent on the metal examined. Equation (12) is valid for non-hydrogenated material; in particular in argon, the resistivity relation vs. temperature for Pd-Ag 21 wt.% was almost linear in the range 25–450 °C, and the α value was about 4.4 × 10^−4^ °C^−1^. In this work, the Pd-Ag resistivity, *ρ°*, at 25 °C in argon was 3.9 × 10^−7^ Ω m.

**Figure 3:**
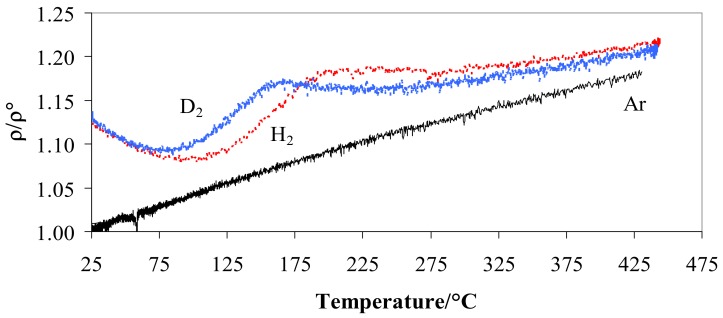
Ratio *ρ/ρ°* vs. temperature for Pd-Ag 21 wt.% in pure hydrogen, deuterium, or argon at 100 kPa.

When the metal is hydrogenated or deuterated, the resistivity depends on the amount and kind of isotopes, gas pressure, and operating temperature [29]. Figure 3 shows the ratio *ρ/ρ°* resistivity vs. temperature at 100 kPa in hydrogen and deuterium compared with the trend in argon. Figure 3 highlights the relationship between *ρ/ρ°* and temperature in hydrogen, deuterium, and argon at a pressure of 100 kPa. 

Figure 3 shows that, at the room temperature, Pd-Ag/H and Pd-Ag/D have a similar resistance (4.4 × 10^−7^ Ω m) with a value greater than 1.13 compared to that of the alloy without the isotopes (3.9 × 10^−7^ Ω m). As the temperature increased, a minimum at T ≈ 85–90 °C was reached for the deuterated alloy, while the hydrogenated material showed the minimum at a higher temperature (95–105 °C). Above 250 °C, the ratio tends to increase slowly for both systems but at the same speed.

### 4.2. Electrical Resistivity vs. Hydrogen Isotopic Content

The lattice imperfections that can increase electrical resistivity are of two kinds: (i) thermal vibrations and ii) impurities and other punctual defects. In a metal alloy like Pd-Ag, lattice vibrations diminish as the temperature decreases: therefore, we assume that their effect on resistivity will modify according to Equation (12).

The total resistivity can be defined for Pd-Ag hydrogenated with H or D as the sum of two terms [51]:

(13) $$\rho =\rho_{T}+\rho_{i}$$

where *ρ_T_* is the resistivity produced solely by the thermal vibration of the Pd-Ag lattice without H atoms; such a parameter increases with temperature as outlined by Equation (12). As regards the second term, *ρ_i_*, it is a complex contribution linked to different effects. The interstitial atoms H (D) influence the electrical resistance of the host metal [50]: their existence in the lattice can be supposed to provide, in part, a second type of lattice imperfection that rises with the amount of H (D) atoms. *ρ_i_* certainly includes a structural resistivity due to impurity scattering of electrons on interstitial protons or vacancies. This term is also connected to electron-phonon scattering due to the vibration of H and D in the lattice: the larger the intensity of vibration at any temperature, the greater the *ρ_i_* is. So, this term depends on several parameters: H (D) concentration into the lattice, temperature, position of H (D) atoms, and their interaction with metal sites. In addition, these different parameters can depend upon each other.

As shown, non-hydrogenated Pd-Ag *ρ_T_* increases with temperature. Combining Equations (12) and (13), we can formalize the contribution of resistivity produced only by the presence of hydrogen or deuterium through the equation:

(14) $$\rho_{i}=\rho -\rho {}^{\circ}\;[1+\alpha \;(T-T{}^{\circ})]$$

**Figure 4:**
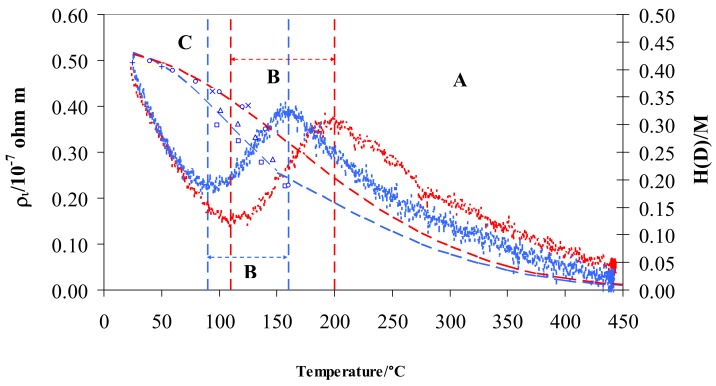
*ρ_i_* vs. temperature for Pd-Ag 21 wt.% in pure hydrogen (red) and deuterium (blue) and relative H/M (red) and D/M (blue) values at 100 kPa. The literature data for D/M: (+) [18], (◯) [21], × [22], (△) [23], (☐) [45] are also shown. Vertical dashed lines show the limits for the different regions A, B, and C in pure hydrogen (red) and deuterium (blue).

From a graphic point of view, we can visualize *ρ_i_* by subtracting the resistivity measured in pure hydrogen or deuterium from that in argon (Figure 4). 

The resistivity behavior exhibits the same S-shaped trend for hydrogen and deuterium. Particularly, the two curves overlap in the range 25–70 °C (contributing 11% of total resistivity) and both curves show a maximum and a minimum with two relevant differences: (i) the minimum and maximum are, respectively, at about 90 and 160 °C in D_2_ and at 110 and 200 °C in H_2_, (ii) maxima of resistivity are the same for D_2_ and H_2_, and (iii) minimum of resistivity in D_2_ is higher than in H_2_.

**Figure 5:**
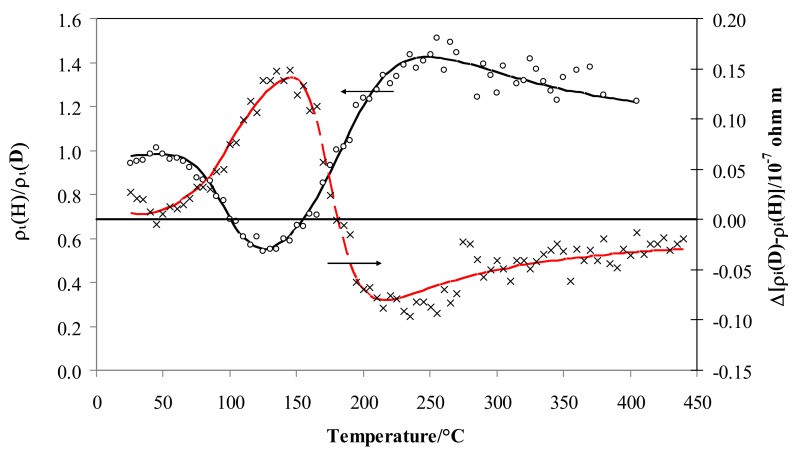
Ratio of *ρ_i_* vs. temperature for Pd-Ag 21 wt.% in pure hydrogen and deuterium (black) and the difference in deuterium less hydrogen (red).

Figure 5 depicts the resistivity isotopic effect expressed as the ratio of the values for hydrogen and deuterium, *ρ_i_*, (left-axis) and their differences (right-axis). The isotopic effect on resistivity expressed by *ρ_i_* is negligible at low temperature (*ρ_i_* is close to 1) but, at about 70 °C, the effect becomes negative (*ρ_i_*^D^ > *ρ_i_*^H^), and after the minimum at 125 °C it increases again, being positive over 160 °C (*ρ_i_*^H^ > *ρ_i_*^D^), achieving a maximum value of 1.41 at about 250 °C and then decreasing above this temperature to a constant value (1.20).

In previous works, the characteristic S-shape for Pd-Ag in hydrogen was explained through the free electrons theory [30,39]: here in this study, the previous analysis is extended to deuterium. In general, three regions were observed when reducing temperature from 450 to 25 °C; (i) region A below 450 °C, where the resistivity, *ρ_i_*, increased until a maximum was reached, (ii) region B, where a decrease of resistivity was observed until a minimum was achieved, and (iii) region C, with a continuous increase of resistivity. The limits in temperature of these three regions are different in hydrogen and deuterium, being related to the H/M and D/M ratio (Figure 4).

The first H or D atoms uploaded into Pd_0.79_-Ag_0.21_ diminish the number of metal valence electrons (*N*) thus raising the resistivity, *ρ_i_* (region A in Figure 4). This process ends when the electrons fill all of the valence band: it occurs at a well-defined value of H/M, calculated so that the sum of H/M + 0.21(Ag) achieves about 40% [40,52,53]. This is confirmed in hydrogen, where we observe the H/M maximum around 0.20 at 200 °C. Furthermore, it is known that, before the d-band is filled, all these hydrogen atoms occupy octahedral interstitial positions. The trend of the resistivity in deuterium was generally quite similar to the hydrogen one, being the differences due to different values of uploading (H/M vs. D/M) along the temperature. The maximum of resistivity in deuterium was shifted at about 160 °C (Figure 4): in fact, at this temperature, D/M reaches a value of about 0.20. The values of maximum resistivity in hydrogen and deuterium are quite similar (about 0.4 × 10^−7^ Ω m): as discussed above, it is noteworthy that these maxima occur with the same occupancy of the isotopes inside the Pd-Ag (H/M and D/M = 0.20). Further absorption of hydrogen (region B in Figure 4) determines a growth of N (number of conduction electrons per unit volume) and, hence, a decreasing of resistivity, *ρ_i_*, down to a minimum (0.15 × 10^−7^ Ω m) with H/M of about 0.34 at 110 °C. We also reach, in deuterium, the minimum of resistivity (but at a higher value of 0.23 × 10^−7^ Ω m) for quite a similar value of uploading (D/M = 0.34) at 90 °C. The electrical resistivity of Pd-Ag, higher in deuterium than in hydrogen at the same ratio (0.34), can be interpreted by the progressive excitation of optical vibration modes [26,48]. In fact, optical vibrations depend on thermal energy, and it is reasonable to expect that the position and energy of D and H in O- and T-sites in the lattice of Pd-Ag is responsible for their different resistivities. 

In part C of Figure 4, resistivity values in both gases increase and overlap below about 70 °C. Above H/M and D/M ratios of 0.34, the scattering effect was predominant in relation to *N* (number of conduction electrons per unit volume): the high concentration of H and D atoms mainly operates as a defect point, thus reducing the electrons’ mean free path and clearly raising the resistivity.

It is interesting to observe that the *ρ_i_* contribution in the range 25–200 °C in both isotopes is similar, from a formal point of view, to that observed for a doped semiconductor where the energy distribution of the carriers varies with temperature (usually near 0 K). In region C (25–70 °C), the resistivity of hydrogenated Pd-Ag decreases with T^−3/2^ (K), following a typical trend for charged impurity scattering in a semiconductor. In the range of temperature from the minimum to the maximum (region B in Figure 4), the resistivity increases with T^3/2^ (K), which is a typical trend for lattice vibrational scattering in semiconductors. The manner in which the two scattering mechanisms vary with T produces the observed minimum. This dependence is only formal because the Pd-Ag is not a semiconductor, but gives an idea of how the electronic conductivity of the alloy is complicated and influenced by the loading of isotopes in relation to temperature.

As the main differences in resistivity were observed in the low temperature range (70–200 °C), a slow heating and cooling ramp of temperature (1 °C/min) was performed in both isotopes in order to verify the effect of exchanging thermal energy on the equilibrium O-sites ↔ T-sites. 

**Figure 6:**
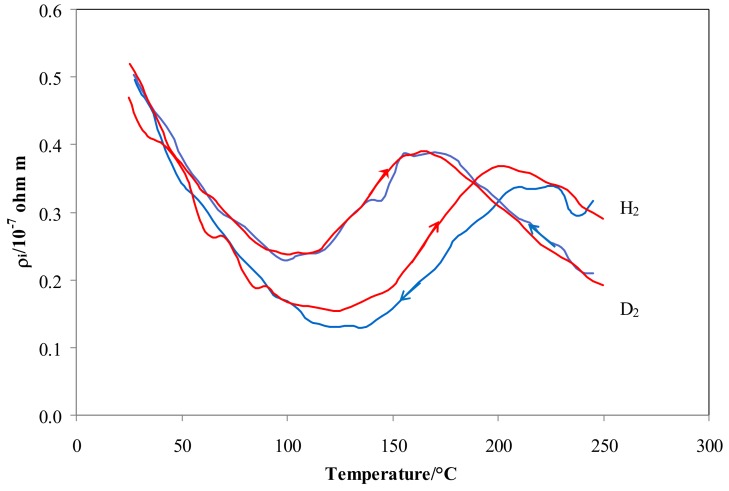
*ρ_i_* vs. temperature for Pd-Ag 21 wt.% in pure hydrogen and deuterium. Red lines refer to heating and blue lines to cooling at 1 °C min^−1^ and 100 kPa.

Figure 6 shows that the results in hydrogen were characterized by a clear hysteresis effect, with a higher resistivity when heated. On the contrary, this hysteresis effect on resistivity does not appear in deuterium. In fact, the difference between |ΔH_S,H_^O^| − |ΔH_S,H_^T^| is higher than that of |ΔH_S,D_^O^| − |ΔH_S,D_^T^|, so that the transition between O-sites and T-sites for hydrogen is characterized by a higher energy barrier. This energy is widely available when moving from high temperature, resulting in lower occupancy and lower resistivity of the cooling curve. On the contrary, moving from low temperature (i.e., heating) the thermal energy needed for the desorption of hydrogen atoms (|ΔH_S,H_^O^| − |ΔH_S,H_^T^|) is available only when higher temperatures (corresponding to these needed energy levels) are achieved. Therefore, in the temperature range of the transition tetrahedral ↔ octahedral, the hydrogen occupancy and resistivity values of the heating curve are higher than those of the cooling curve at a given temperature. Since the difference, |ΔH_S,H_^O^| − |ΔH_S,H_^T^, is small, the resistivity curve of deuterium does not exhibit a noticeable hysteresis effect. 

## 5. Conclusions

The experimental work was aimed at measuring the resistivity of Pd-Ag in hydrogen and deuterium at a pressure of 100 kPa and in a temperature range of 25–450 °C. The results showed that electrical resistivity is dependent on the ratio of H/M and D/M. The typical S-shaped behavior of resistivity has been modeled via the free electron theory. The interaction of conduction electrons with hydrogen and deuterium in the Pd-Ag lattice gives rise to different effects:

- The loading of hydrogen and deuterium atoms at low H/M ratios reduces the number of conduction electrons, thereby increasing resistivity,
- Above a well-marked and similar H/M and D/M ratio (about 0.20), the further absorption of isotopes leads to an increase in the conduction electrons, thus reducing the resistivity,
- Further increasing the H/M or D/M ratio beyond a certain value (about 0.34), the electron scattering against the large number of isotope atoms (operating as reticular defects) becomes prevalent, and the resistivity grows very rapidly.

Cooling or heating at a slow rate between 25–250 °C evidenced a hysteresis effect of resistivity in hydrogen that can be explained by the different energy levels of hydrogen atoms in O-sites and T-sites. Future experiments on hydrogen absorption into Pd-Ag will be addressed to confirm the hysteresis for the curve of H/M vs. the temperature via Sievert’s law or gravimetric methods.
